# Rapid scoring of genes in microbial pan-genome-wide association studies with Scoary

**DOI:** 10.1186/s13059-016-1108-8

**Published:** 2016-11-25

**Authors:** Ola Brynildsrud, Jon Bohlin, Lonneke Scheffer, Vegard Eldholm

**Affiliations:** 1 Domain of Infectious Disease Control and Environmental Health, Norwegian Institute of Public Health, Oslo, Norway; 2 Hanze University of Applied Sciences, Groningen, The Netherlands

**Keywords:** Pan-genome, Accessory genome, Annotation, Prokaryote, Genomics, Whole-genome sequencing (WGS), Next-generation sequencing (NGS), Python, Bacteria, Genome-wide association studies (GWAS), Association

## Abstract

Genome-wide association studies (GWAS) have become indispensable in human medicine and genomics, but very few have been carried out on bacteria. Here we introduce Scoary, an ultra-fast, easy-to-use, and widely applicable software tool that scores the components of the pan-genome for associations to observed phenotypic traits while accounting for population stratification, with minimal assumptions about evolutionary processes. We call our approach pan-GWAS to distinguish it from traditional, single nucleotide polymorphism (SNP)-based GWAS. Scoary is implemented in Python and is available under an open source GPLv3 license at https://github.com/AdmiralenOla/Scoary.

**Electronic supplementary material:**

The online version of this article (doi:10.1186/s13059-016-1108-8) contains supplementary material, which is available to authorized users.

## Background

Whole-genome sequencing (WGS) of bacteria is routinely performed in many laboratories all over the world, producing enormous amounts of accurate genome data, the majority of which is poorly understood. Genome-wide association studies (GWAS) have in human medicine and genomics become a workhorse for linking genetic variants in a population with observed phenotypes, but bacterial GWAS have only very recently started to emerge [1–9]. These studies have focused on clinically relevant phenotypes, such as virulence and antibiotic resistance, but the methodology has potential for understanding causal determinants of phenotypes that are relevant to industry and environmental purposes as well [10].

Bacterial GWAS cannot directly adopt eukaryotic methods due to a number of important idiosyncrasies of bacterial evolution and the bacterial genome. Notably, the entire bacterial genome is considered to be in linkage disequilibrium, chromosomes and plasmids recombine internally and across phyla, and population samples are often stratified into multiple clusters of clonally related isolates. However, one advantage is that some mutations, at least clinically relevant ones, have high penetrance and are subject to high selective pressure. This makes causal links easier to establish, i.e. smaller sample sizes can result in statistically significant associations.

Many bacterial phenotypes can be linked to the presence or absence of particular genes that are inherited through descent or acquired through lateral gene transfer. The full complement of all genes among a set of genomes is referred to as the pan-genome [11, 12]. The construction of the pan-genome is a nucleotide polymorphism (NP)-hard problem that traditionally has taken days to weeks to perform and which for large datasets have simply been impossible. Recent algorithmic advances have however made the construction of a pan-genome both rapid (now taking merely hours) and scalable [13]. These advances will permit large-scale adoption of GWAS-methodology in bacteria, on the condition that accessible, powerful, and user-friendly software is developed.

Here we present and benchmark Scoary, an easy-to-use, ultra-fast tool for studying the association between pan-genome genes presence or absence and observed phenotypes. We term the method “pan-GWAS” to distinguish it from traditional SNP-based GWAS. Each candidate gene in the accessory genome is sequentially scored according to its apparent correlation to predefined traits. Genes that pass the initial screening are re-analyzed while incorporating information on the phylogenetic structure of the sample. This correction step makes minimal assumptions about evolutionary processes and directly infers population structure from the input data. This ensures reproducibility as well as accessibility for users with limited bioinformatics and population genetics skills, as they need not experiment with ill-informed mutation rate parameters or even inform the program about population structure at all. In order to ensure the validity of results, Scoary implements a post-hoc label-switching permutation test. As few as 20 samples can in some cases be enough to implicate a causal gene, which we demonstrate by applying our method to study linezolid resistance in *Staphylococcus epidermidis*. We further report the power of our software over a range of sample sizes by subsampling of a large *Streptococcus pneumoniae* dataset, demonstrating high power in sample sizes larger than 100. Finally, we investigate how gene penetrance and sample size affect the power to detect causal genes using simulated data and benchmark our program against the program PLINK [14], which has been widely adopted in human, SNP-based GWAS. We have named our gene-scoring program Scoary in homage to the pan-genome construction software Roary [13].

## Results

### General description

Scoary is implemented as a standalone python script with SciPy [15] as it is only non-standard dependency. It was designed with three goals in mind: (1) it should be intuitive, platform-independent, and simple to use and should give the user easily understandable results. A graphical user interface wrapper is available for maximized user-friendliness. (2) It should be able to work with typical experiment sample sizes, i.e. tens or hundreds rather than thousands of genomes. (3) It should be quick, allowing a user to screen rapidly a genome collection in a few minutes rather than days or weeks.

To enable the efficient execution of these computationally demanding tasks, they are performed by Scoary in a multiprocessing environment. Using 50 samples with a total of 23,133 gene families, we fully analyzed every gene for associations to three different traits in 7 s when tested on a standard desktop computer with 4 CPU cores and 8 GB RAM. The internal algorithm makes very few assumptions and, as such, does not require extensive parameter estimation. Two files are needed as input: a genotype (typically with gene presence/absence) matrix and a trait file. The former is created by Roary and can be fed directly to Scoary. The latter has to be created by the user and is a simple matrix (e.g. Excel/Calc sheet) describing the phenotype status for each strain. Traits must be binary, but more complex data (e.g. ordinal traits such as pathogenicity or semi-continuous traits such as MIC values) can also be used by breaking observations into dummy categories.

### Program fundamentals

**Fig. 1:**
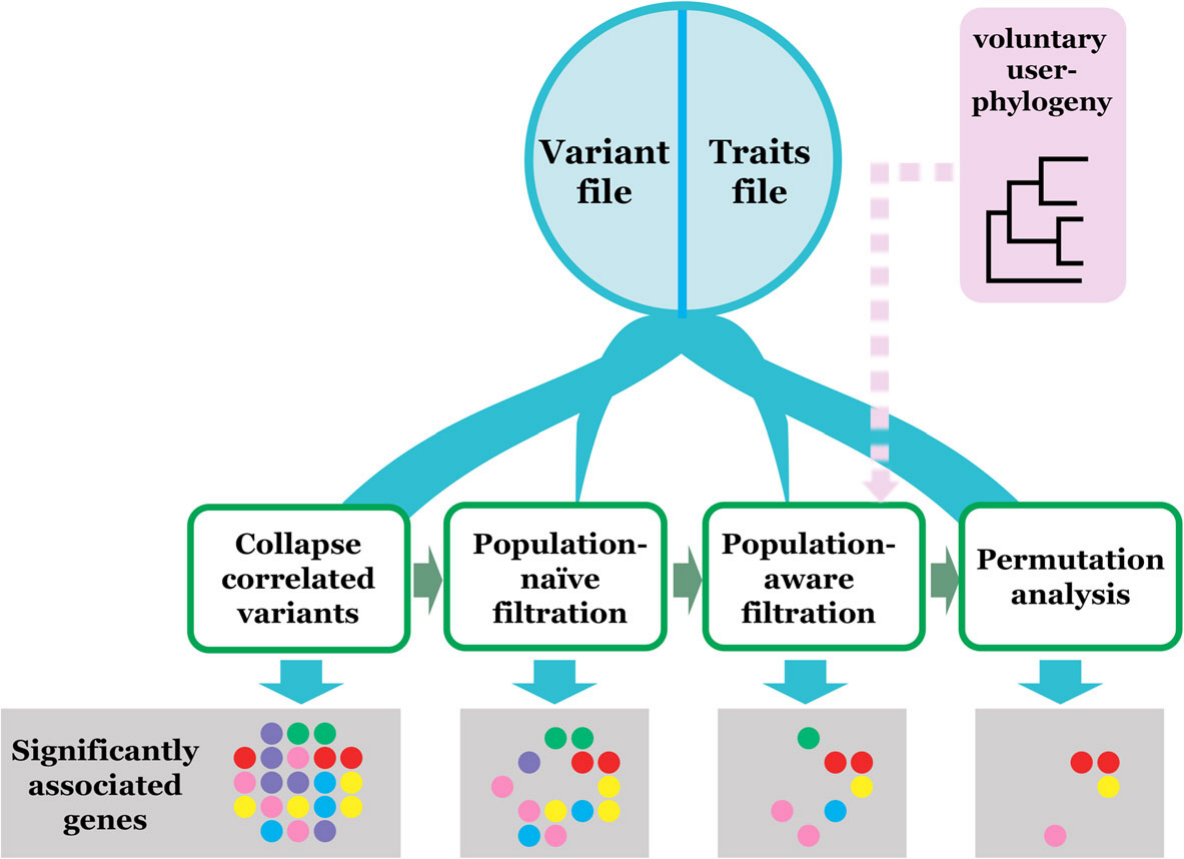
Overview of Scoary workflow. The main input files are one genotype and one phenotype matrix and optionally a phylogenetic tree that will define sample genealogy. If the latter is not provided it is calculated internally through the isolate Hamming distances of the input genotype file. Each candidate variant goes through a set of filtration steps, the thresholds for each set by the user. Fewer and fewer candidate variants will be left to analyze as the computational complexity of operations increase. Variants that pass all filters are returned as results

An overview of the Scoary workflow can be seen in Fig. 1. The main idea is that candidate variants can be passed through a series of filters. Variants that fail a filter are discarded, while those that pass all filters are returned as results. The filtration proceeds from computationally cheap towards computationally expensive operations, thus ensuring that resources are not wasted on variants that are clearly not associated with the phenotype. Each filter can be turned on and off and filter thresholds set individually.

For each phenotype supplied via columns in the traits file, Scoary does the following: first, correlated genotype variants are collapsed. Plasmid genes, for example, are typically inherited together rather than as individual units and Scoary will collapse these genes into a single unit. Each candidate variant then receives its own null hypothesis of no association to the trait. As a first and optional filtration step, a Fisher’s exact test is performed on each variant in a population-agnostic manner. This happens as follows: A 2 × 2 table is created, the levels being positive or negative for the trait and gene, respectively, and the number of isolates in each cell is then counted. Variants that are present in every input isolate are excluded since they provide no information as to which variants are responsible for a trait that is differentially distributed in the sample. Similarly, variants that are not present in any input isolates are excluded (only relevant when analyzing isolate subsets).

Since the program investigates a potentially huge number of null hypotheses, additional filtration can be specified using the Bonferroni and Benjamini–Hochberg adjustments [16, 17] to correct for multiple comparisons.

### Population structure correction

**Fig. 2:**
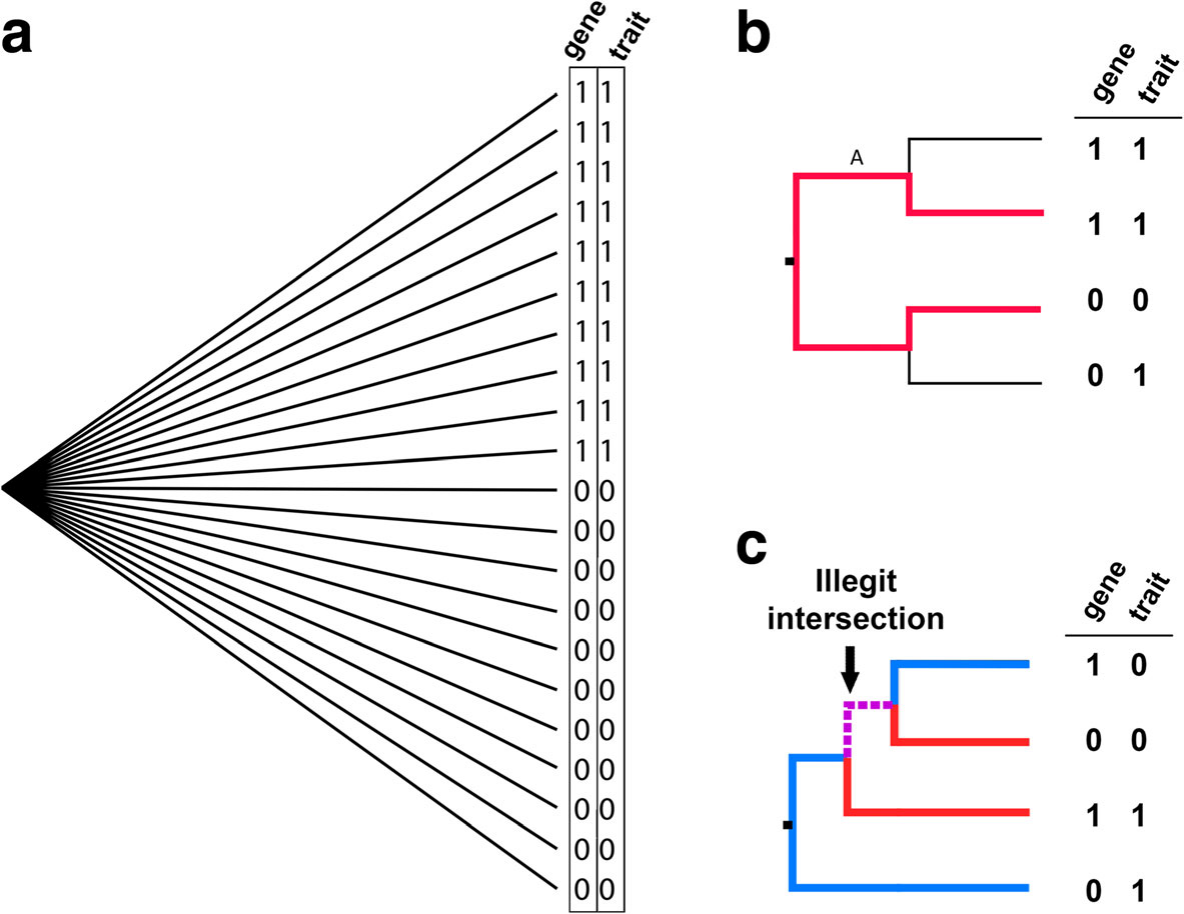
Pairwise comparisons introduction. **a** *Star tree*, all isolates equidistantly related. In this scenario, each isolate has a random and independently distributed probability of exhibiting each state and Fisher’s exact test is appropriate. **b** In *non-star trees*, the probability of exhibiting each state is confounded by the population structure, in this case meaning the evolutionary history of the sample. An appropriate way of handling this is shifting focus towards evolutionary transitions, as in the pairwise comparisons algorithm. This figure shows the basic idea of a contrasting pair. This *tree* has a maximum number of 1 non-intersecting, contrasting pairs, a 1–1|0–0 pair. **c** An illegit pairing. While the two middle isolates and the top and the bottom isolates are both able to form a contrasting pair, a single picking cannot pick both pairs as they would intersect (shared branch shown stapled in *purple*). Thus, the maximum number of contrasting pairs in this tree is 1. The “best” picking is the *red* pair (1–1|0–0), which supports gene = 1 - > trait = 1 and the “worst” picking is the *blue* pair (1–0|0–1), which supports gene = 0 - > trait = 1. The associated *p* value is equal to 1.0 in either case

**Fig. 3:**
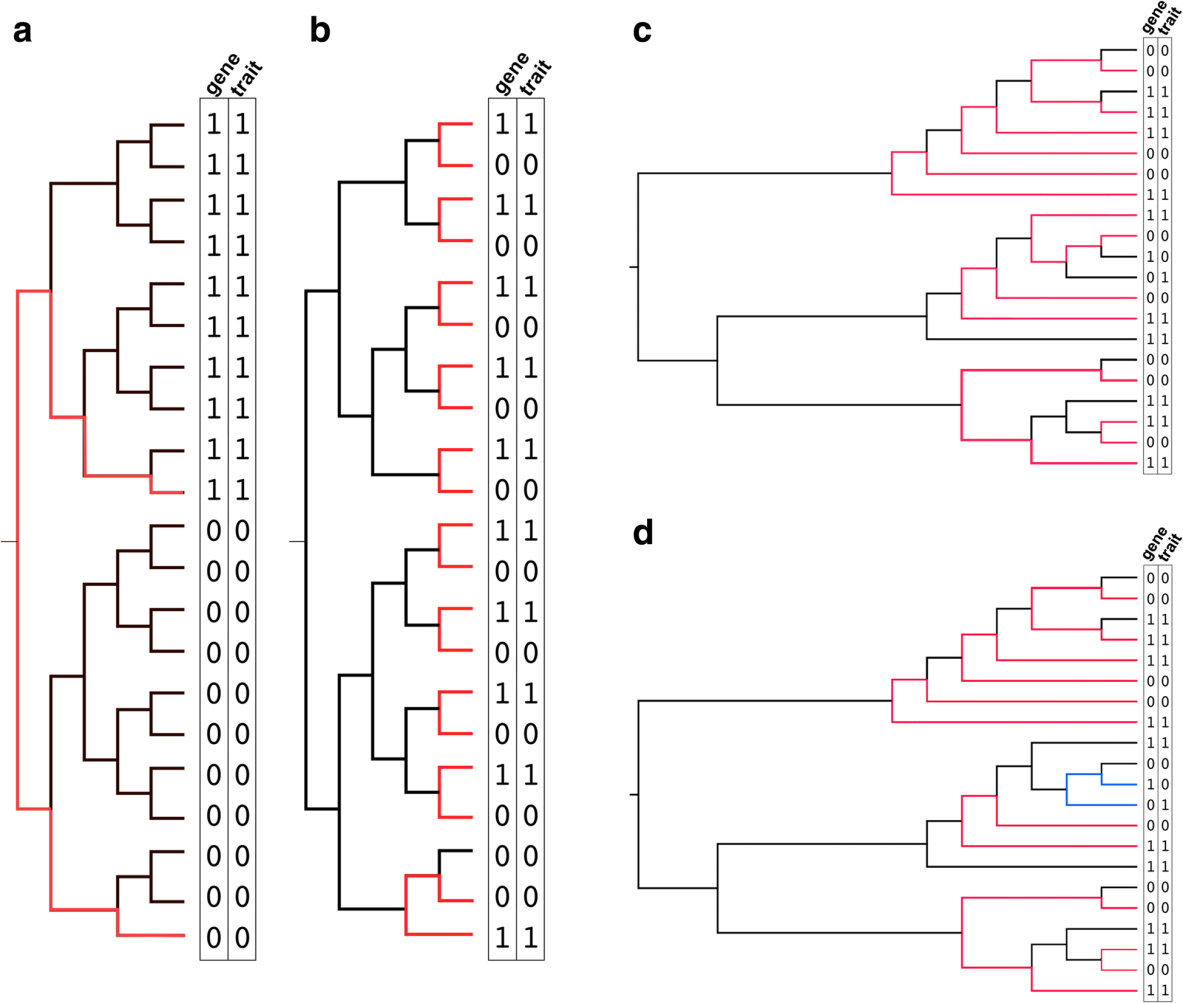
Pairwise comparisons examples. **a** Fisher’s exact test for this sample would be highly significant (*p* = 2.8E-6); however, upon inspection of the tree it becomes clear that there are lineage-specific interdependencies which is a violation of the randomness model implicit in Fisher’s test. The top samples, which display 1–1 are more closely related to each other than the bottom samples, which display 0–0, and vice versa. The most parsimonious scenario is a single introduction (or loss) of the gene and the trait on the root branch. This is illustrated by the pairwise comparisons algorithm, which can find a maximum of 1 contrasting pair (0–0|1–1). **b** Contrast this to (**a**). This tree has a maximum of ten contrasting pairs, all 0–0|1–1, which indicates a minimum of ten transitions between 0–0 and 1–1 in the evolutionary history of the sample. In this situation, we should be more convinced that there is a true association between this gene and the trait. The associated *p* value of the binomial test (the statistical test in the pairwise comparisons algorithm) would be 0.0019. Note that the gene-trait matrix is identical to the one in (**a**), only shuffled to correspond to tree leaves. **c** Tree with a maximum number of 7 non-intersecting, contrasting pairs. In this picking, all pairs are 1–1|0–0, indicating a binomial test *p* value of 0.015, a “best” picking of pairs. **d** Another picking of 7 contrasting pairs from of the tree in (**c**), but this set of pairs includes a 1–0|0–1 pair, corresponding to a *p* value of 0.125. This represents a “worst” picking of pairs from the tree. Thus, the full range of pairwise comparison *p* values for the gene-trait-phylogeny combination in (**c**) and (**d**) would be 0.015–0.125

An assumption in Fisher’s test is that all isolates have a random and independently distributed probability for exhibiting each state (Fig. 2a). As such, it is in most real populations unsuitable for causal inference because the probability of exhibiting each state is dependent on the population structure. To control spurious associations from stratified populations, Scoary therefore implements the pairwise comparisons algorithm [18, 19]. This requires a phylogenetic tree, which can be supplied by the user or calculated internally by Scoary from the Hamming distances in the genotype matrix. The idea of pairwise comparisons is to find the maximum number of phylogenetically non-intersecting pairs of isolates that contrast in the state of both genotype and phenotype (See Fig. 2b and c). By doing this, focus is shifted towards evolutionary transitions as the unit of concern rather than terminal isolates [20]. Finding the maximal number of contrasting pairs effectively counts the minimum number of independent co-emergences of a given gene-trait combination in the evolutionary history of the sample population (as represented by the tree), thus avoiding the problem of “pseudoreplication of lineage-specific factors” [21]. It is particularly effective for controlling for bias from clonal sampling schemes (see Additional file 1). As an example, consider Fig. 3a and b: the gene-trait matrix is identical in these two examples and Fisher’s exact test results in identical *p* values (2.8E-6) for both matrices. In Fig. 3a, however, it is clear that the apparent association could be caused by a single evolutionary transition on the root branch, whereas Fig. 3b requires ten independent transitions. Thus, the scenario in Fig. 3b clearly represents strong evidence for a causal association between gene and phenotype contrary to the scenario outlined in Fig. 3a.

If the null hypothesis is true, there should be approximately equally as many 1–1|0–0 pairs as 1–0|0–1 pairs (following a genotype-phenotype annotation, e.g. 1–1 means an isolate that is positive for both the genotype variant and the phenotype) [18, 22]. Hence, under the null hypothesis, the phenotype has been randomly assigned as either 0 or 1 with *p* = 0.5 for each possible outcome irrespective of the genotype. Pairs that contrast in one variable but not the other (e.g. 1–0|0–0) are not considered informative [18].

This method allows probabilities of evolutionary change to vary throughout the tree, but does not actually require that these probabilities or an explicit evolutionary model be specified. There are usually many possible maximal pairings and among these some might provide more support for a significant association than others. Given a maximal number of pairs that contrast in both the gene and the trait states, Scoary calculates the maximum and minimum number of pairs that support an association. In the following, we will assign the terms “best” and “worst” pairings for these respective scenarios. Figure 3c and d illustrate this. In a tree with a maximum of seven possible contrasting pairs, a best possible pairing might be seven 1–1|0–0 pairs (Fig. 3c) and a worst might be six 1–1|0–0 and one 1–0|0–1 pair (Fig. 3d). *P* values corresponding to the best and worst scenarios are calculated with binomial tests and both values are reported. Using our example above, the best pairing (7/7 1–1|0–0 pairs) would correspond to a *p* value of 0.015 and the worst pairing (6/7 1–1|0–0 pairs and 1/7 1–0|0–1) would correspond to a *p* value of 0.125. Note that it is not quite clear how to interpret such a *p* value range as some possible pairings might be better than others (e.g. many phylogenetically “shallow” pairs) and the number of possible pairings associated with one scenario could be much higher than the number of possible pairings associated with the other [19]. A conservative approach would be to require that both the best and the worst possible *p* values are less than the predetermined alpha in order to score the association as significant.

### Permutation

An additional test implemented by Scoary is that of label-switching permutations. This is achieved by making random permutations of the phenotype data and calculating the associated test statistic (maximum number of 1–1|0–0 pairs divided by maximum number of pairs) for each permutation. The permutation statistics are sampled under a situation where the null hypothesis is true, since any association between the genotype and phenotype is broken by the random sampling. If N is the number of permutations and r is the number of test statistics observed to be higher or equal to the unpermuted statistic, the empirical *p* value is returned as (r + 1)/(N + 1) [23]. Depending on the number of permutations, this can be a laborious procedure and the permutation procedure is therefore realized through the use of multiple CPU processes simultaneously.

### Output

The output of Scoary is a single list of significant genes per trait. Each trait results file reports the highest scoring genes, i.e. those genes that were most associated (either positively or negatively) with the trait, sorted by *p* values. The output can be controlled by a number of optional parameters, such as max number of hits and *p* value cutoff.

#### Scoary performance

##### Linezolid resistance in Staphylococcus epidermidis

Linezolid (LZD) is an oxazolidinone-type antibiotic used to combat Gram-positive bacteria such as methicillin-resistant *Staphylococcus aureus* (MRSA) and methicillin-resistant coagulase-negative staphylococci (CoNS) [24]. LZD-resistant staphylococci are uncommon, but have been reported worldwide [25].

We applied Scoary to identify genes associated with high-level resistance to LZD in *Staphylococcus epidermidis* by applying the algorithm to a set of 21 isolates for which public WGS data and LZD minimum inhibitory concentration (MIC) values had been published [26]. MIC values were dichotomized into ≥ 128 and < 128 μg/mL.

Scoary correctly predicted the well-known LZD resistance gene *cfr,* as significantly associated with high-level resistance to linezolid, although the high end of the pairwise comparison p value range exceeded 0.05 (Table 1). Additionally, Scoary identified two other plasmid-associated genes (*pinE, cueR*) that were associated with the trait, which were significant (p < = 0.05) after pairwise comparisons. Fisher’s test additionally identified several other genes as significant prior to population-aware analysis, but upon inspection of the population distribution these were found to be lineage-specific effects and were concordantly not reported as significant by the pairwise comparisons test.

**Table 1:** Highest-ranking genes for association with LZD resistance MICs ≥ 128 μg/mL

Gene	Comment	OR	*p*	Pairwise p	Accession	Reference
*cfr*	*cfr*-family 23S ribosomal methyltransferase	18	*0.0089*	*0.0156* - 0.1093	AGJ70604	[33]
*pinE*	DNA recombinase (pLRSA417-associated)	Inf	*0.0038*	*0.03125 - 0.03125*	EXP54287	[34]
*cueR*	HTH-type transcriptional regulator	Inf	*0.0123*	*0.03125 - 0.03125*	AJM87290	[34]
Merged^a^	Presumed plasmid genes	Inf	*0.0123*	0.0625 - 0.0625	-	-
*blaR1*	Beta-lactamase regulatory protein	0.067	*0.0237*	0.0625 - 0.3125	WP_001096374	[35]
Hypo.	Hypothetical protein	Inf	*0.0350*	0.0625 - 0.0625	-	-

*P* values < = 0.05 in italics

^a^YolD-like protein, TrbC/VIRB2-family protein, seven hypothetical proteins

*OR* odds ratio, *p* naïve *p* value, *Pairwise p* range of *p* values from the pairwise comparisons, *Emp p* empirical *p* value after 1000 permutations

#### Scoary performance

##### Power studies on a large Streptococcus pneumoniae dataset

Erythromycin is a macrolide-type antibiotic compound used against a wide range of bacterial infections. In *Streptococcus pneumoniae*, erythromycin resistance is bestowed by the presence of the *erm* gene [27]. To estimate Scoary’s power under a range of different sample sizes, we used previously published WGS data from a large study on *S. pneumoniae* sampled from a refugee camp in Thailand [1] with erythromycin drug susceptibility test results. The advantage of using a real dataset rather than simulated data is that we can be sure that the trait distribution and phylogenetic relationships between isolates in any subsample mimic what one might get in a real study of sample size N. We first ran Scoary on the full dataset of 3085 isolates to verify that the *erm* gene was significantly associated with resistance.

From the total dataset of 3085 isolates, we tested sample sizes (N) in the range of 20–200 and for each sample size N we sampled ten random subsets of N isolates. On each of these datasets, we ran Scoary with three different filtration cutoffs: (1) population-naïve Fisher’s exact test p value < 0.05; (2) same as (1), but with the additional requirement that the entire range of pairwise comparisons p values were < 0.05; (3) same as (2), but with the additional requirement that the empirical p values of the variant after 1000 permutations were ≤ 0.05. Table 2 shows the power under each filtration scenario (for simplicity, only every third N in our experiment is shown). Here power is used as the proportion of runs in which the specific gene unit was reported as significant. Note that the *erm* gene was relatively rare in the sample (prevalent in 331 samples, 10.7% of the sample) and the subsets were randomly selected without regard for genotype or phenotype status. As a general rule, equal proportions of each genotype/phenotype variant would be associated with higher power, while lower power than that demonstrated here would be attained in a sample dominated by one genotype/phenotype status (*e.g.* very low prevalence of gene/phenotype in sample) (see Additional file 2).

**Table 2:** Power under a range of sample sizes and filtration scenarios. Here power is defined as the proportion of replicates out of ten in which the *erm* gene was found to be associated with erythromycin resistance. Scenario 1: following Fisher’s exact test; Scenario 2: same as 1 but added population size correction (*p* value range < 0.05); Scenario 3: Same as 2 but with additional requirement that the empirical *p* values after 1000 permutations were < 0.05

Sample size (N)	Scenario 1	Scenario 2	Scenario 3
20	0.2	0.0	0.0
50	1.0	0.0	0.0
80	1.0	0.2	0.2
110	1.0	0.8	0.4
140	1.0	0.8	0.7
170	1.0	1.0	0.6
200	1.0	0.9	0.8

#### Scoary performance

##### Power tests on simulated data

In order to understand how Scoary is affected by the phenotypic penetrance of causal genes under various sample sizes, we additionally tested the power of Scoary on simulated data. The simulated genomes were created with a custom script (see “Methods”). Briefly, the process starts with a single root genome and at each time point all currently existing genomes are randomly allowed to mutate (changing the gene content) and have a probability of branching (creating an additional isolate) until a desired target number of isolates exists (for details, see “Methods”). The root genome starts with 3000 genes present and 6000 genes absent. (The sum (9000) represents the full complement of attainable genes.) One gene was set as the causal gene and an isolate may acquire or lose this or any other gene at any time. In addition to sample size, the script allowed us to experiment with the penetrance of the gene (here used as the chance of acquiring/losing a phenotype at the same time as the causal gene is acquired/lost) as well as the gene recombination rate (rate of acquisition/loss).

Our results demonstrate that Scoary performs remarkably very well at small sample sizes. At 90% phenotypic penetrance, the mean F1 score [28] (see “Methods”) is above 0.7 in sample sizes that are 50 or greater and the recall rate is also at least 70%. If the penetrance drops to 75%, a sample size of at least 150 seems to be required in order to ensure equally high F1 scores, however the recall rate remains relatively high (80%) at a sample size of 100, but at the cost of a significantly higher number of false positives.

### Comparison with PLINK

**Fig. 4:**
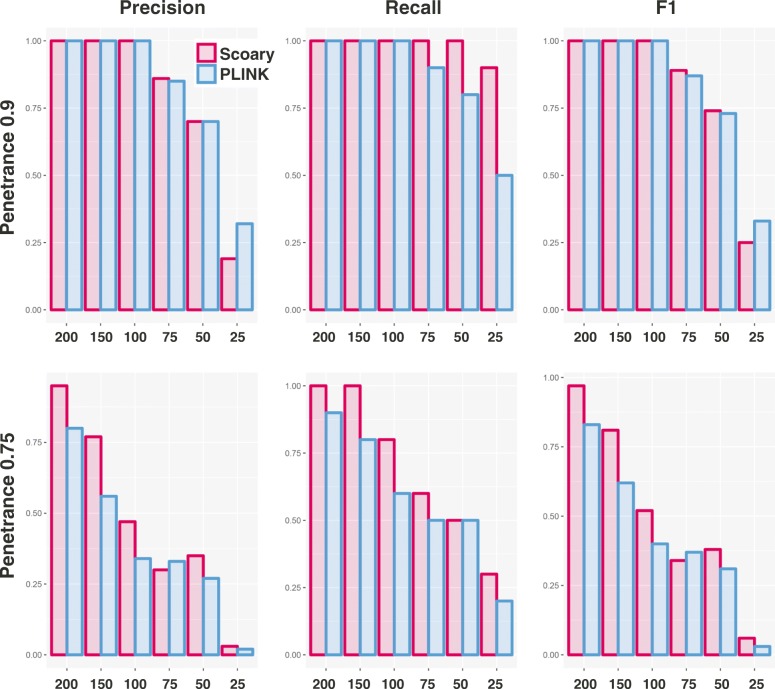
Comparison between Scoary and PLINK. The graphs show precision, recall, and average F1 scores by sample size and causal gene penetrance

Although originally designed for human GWAS, a few microbial association studies [2, 29] have used the software PLINK [14]. Note that PLINK was made for SNP-type genotypic variation rather than gene presence/absence and that it assumes diploidy and is thus from a purely theoretical perspective not appropriate for bacterial data. In practice, however, it performs well enough: we ran PLINK on all simulated datasets that were previously used for benchmarking Scoary and our results (Fig. 4) indicate that Scoary outperforms PLINK in 7/12 comparisons, performs equally well in three comparisons, and slightly worse in two comparisons (as measured by the parameter-average F1 score).

## Conclusion

We have developed a method to score the components of a pan-genome for associations to traits. Our method is implemented in an open-source tool named Scoary and can be run on all modern computers with python and SciPy installed.

## Methods

### Linezolid resistance in *Staphylococcus epidermidis*

Sequence data (accession number SRP039360) was downloaded from NCBI Sequence Read Archive (SRA), subsampled to coverage 50 with khmer 2.0 [30], and assembled with mismatch correction in SPAdes 3.6.2 [31]. Contigs with length < 500 and coverage < 2.0 were removed by an in-house script. Assemblies were annotated with PROKKA 1.11 [32] using the *Staphylococcus* genus database. The pan-genome was constructed using Roary 3.4.2 [13]. Scoary 1.6.3 was run with the default options.

### Antibiotic resistance in *Streptococcus pneumoniae*

Sequences that were previously assembled by Chewapreecha et al. [1] as well as resistance metadata were downloaded from ftp://ftp.sanger.ac.uk/pub/pathogens/Streptococcus/pneumoniae/. These were annotated with PROKKA 1.11. The pan-genome was constructed using Roary 3.4.2. We created a custom script that randomly sampled a predetermined number of isolates (range of 20–200, with a step of 10) from the full set of 3085 and ran Scoary 1.6.3 with the --restrict_to parameter. This was done ten times for each sample size and replicated for each filtration scenario 1, 2, and 3, as described in the Results section.

### Simulation of genomes and benchmarking

We created a custom script (available at https://github.com/AdmiralenOla/Simulate_pan_genome) that worked as follows. Evolution starts with a single root genome with 3000 genes and an additional set of 6000 genes that could possibly be acquired (representing the entire pan-genome). At the onset of evolution, each genome in the collection independently acquires and loses genes at gene-specific rates sampled from a uniform distribution (0.0–0.01 per time). At each mutation event, each genome also has a chance to duplicate itself (representing a branching event), after which the duplicate is added to the collection. When the total number of genomes in the collection reaches a predetermined desired sample size, evolution stops and the pan-genome is returned in a file similar to Roary’s gene presence/absence file. Two parameters can be specified by the user: (1) the desired number of samples; and (2) the causal gene penetrance, defined here as the probability of also acquiring/losing the studied phenotype simultaneously to an acquisition/loss of causal gene event. We ran this script ten times for each possible combination of the following parameters: sample size: 25, 50, 75, 100, 150, 200; penetrance: 90, 75. The *p* value cutoff was set based on initial simulation rounds. The F1 score was calculated as the harmonic mean of the precision and recall rate, here defined as follows: Precision = Number of true positives divided by the number of positives returned. Recall (sensitivity) = Fraction of true positives that are actually identified as positives. The mean F1 score is reported for each parameter combination.

Stratified-population association analysis in PLINK v 1.07 was ran by first clustering groups (−−cluster flag), requiring at least five isolates in each group and a pairwise population concordance (ppc) significance of less than 0.05 for grouping. Association analysis was carried out with the Cochran-Mantel-Haenszel association statistic (−−mh flag) to condition on the clusters. We used the Benjamini-Yekutieli adjusted *p* values for significance and the *p* value cutoff was chosen post hoc so that the F1 score would be maximized.

## Availability and requirements

Scoary is implemented in Python (2.7+ and 3.x) and is available under an open source GPLv3 license at https://github.com/AdmiralenOla/Scoary. Its only non-standard dependency is SciPy [15] version 0.16+.

## Additional files

Additional file 1: A case for pairwise comparisons demonstrating how it avoids inflation of false positive results from clonal sampling schemes. (DOCX 18 kb)
Additional file 2: Power test on rare variants, demonstrating how power is related to the baseline prevalence of variants in the sampled population. (DOCX 26 kb)

## Abbreviations

GWAS: Genome-wide association study
LZD: Linezolid
MIC: Minimum inhibitory Concentration
OR: Odds ratio
