# Erratum to: Rapid scoring of genes in microbial pan-genome-wide association studies with Scoary

**DOI:** 10.1186/s13059-016-1132-8

**Published:** 2016-12-19

**Authors:** Ola Brynildsrud, Jon Bohlin, Lonneke Scheffer, Vegard Eldholm

**Affiliations:** 1 Domain of Infectious Disease Control and Environmental Health, Norwegian Institute of Public Health, Oslo, Norway; 2 Hanze University of Applied Sciences, Groningen, The Netherlands

## Erratum

After the publication of this work [1] it was noticed in the third paragraph of the ‘Background’ where it reads:

The construction of the pan-genome is a nucleotide polymorphism (NP)-hard problem that traditionally has taken days to weeks to perform and which for large datasets have simply been impossible.

(NP) does not mean ‘nucleotide polymorphism’. This has now been removed from the sentence.

The original version of this article has been updated.

The publisher apologises for these errors.
