# Supplementary material for: Rapid scoring of genes in microbial pan-genome-wide association studies with Scoary
Source: Genome Biol. 2016 Nov 25;17:238. doi: 10.1186/s13059-016-1108-8 (PMC5124306; doi:10.1186/s13059-016-1108-8)
Supplement: Additional file 1: — A case for pairwise comparisons demonstrating how it avoids inflation of false positive results from clonal sampling schemes. (DOCX 18 kb) [file 13059_2016_1108_MOESM1_ESM.docx]

***Scoary Performance:*** *Pairwise comparisons to control clonal sampling.*

To further the case for pairwise comparisons as a way of controlling spurious results, we verify that it, as opposed to naïve tests such as Fisher’s exact test or other methods that don’t control for pseudo-replication of lineage-specific factors, is unaffected by clonal sampling.

Commonly in WGS experiments, the sample phylogeny is not known *a priori*. In many cases, a large number of samples may belong to a relatively clonal lineage, while others are more distantly related. The clonal sampling can drive results if association analysis is performed in a population-agnostic manner. As an easy example, consider a situation where a gene –trait association is *near* significance by Fisher’s exact test, demonstrated by the following 2x2 table:

|  | Gene positive | Gene negative |
| --- | --- | --- |
| Trait positive | 5 | 1 |
| Trait negative | 1 | 4 |

In this case, Fisher’s exact test gives a p-value of 0.08. However, note that we if we take one of the gene- and trait-negative isolates and sample it two more times, the p-value drops to 0.03. Thus, by simply sampling the same or a highly related isolate we have now reached a state where it looks like there is a true association between this gene and the trait.

Contrarily, the pairwise comparisons p-value would be unchanged by this type of sampling regardless of how many extra isolates are included, since this does not inflate the number of evolutionary transitions in the tree.

We can show this by again using the *Staphylococcus epidermidis* genomes and the data on linezolid resistance [1]. We inflated the sample number ten-fold to 210 by clonal re-sampling. For each original isolate, we randomly let each gene have a 99.5% chance of appearing in the clonally re-sampled isolate as well.

In this run, 1136 genes have Fisher’s test p-values lower than 0.05, 1022 after Benjamini-Hochberg correction. Only the *cfr* and *cueR* genes have pairwise comparison p-values lower than 0.05.

The results demonstrate that while the p-values from Fisher’s exact test indicate a high degree of association, the pairwise comparisons based p-values are virtually unaffected. We conclude that the method is appropriate to control false positive results from clonal sampling.

The data underlying this analysis is publicly available at https://github.com/AdmiralenOla/datasets

1. Tewhey R, Gu B, Kelesidis T, Charlton C, Bobenchik A, Hindler J, Schork NJ, Humphries RM: **Mechanisms of linezolid resistance among coagulase-negative staphylococci determined by whole-genome sequencing**. *MBio* 2014, **5**(3):e00894-00814.
