# Supplementary material for: Rapid scoring of genes in microbial pan-genome-wide association studies with Scoary
Source: Genome Biol. 2016 Nov 25;17:238. doi: 10.1186/s13059-016-1108-8 (PMC5124306; doi:10.1186/s13059-016-1108-8)
Supplement: Additional file 2: — Power test on rare variants, demonstrating how power is related to the baseline prevalence of variants in the sampled population. (DOCX 26 kb) [file 13059_2016_1108_MOESM2_ESM.docx]

***Scoary Performance:*** *Estimating power over a range of variant prevalences*

One major factor in determining the power to detect causally associated genes is the population prevalence of a particular variant. To test this we again used the 3,085 *S. pneumoniae* sampled from a refugee camp in Thailand [1] with information about resistance towards beta-lactam antibiotics. Penicillin targets the synthesis of peptidoglycan, the major component of the bacterial cell wall. In *Streptococcus pneumoniae*, the penicillin-binding proteins play essential roles in peptidoglycan synthesis and are also the targets of penicillin. The data was dichotomized into susceptible versus intermediate and resistant. We first ran Scoary on the full dataset of 3,085 isolates and selected genes with a pairwise comparisons p-value range fully below 1E-7 as true positives. This resulted in a total of 7 genes that could be collapsed into 5 independent units, detailed in Supplementary Table 1. Note that these genes refer to specific variants. Some of the genes (*gpsB*, *cbpC*) are known as essential genes [2-4], but with multiple isoforms, some of which seem to be associated with resistance.

| Supplementary Table 1 Five independent gene units found to be associated with resistance towards beta-lactams in the full set of 3,085 *S. pneumoniae*. Genes with perfect or near-perfect correlation has been collapsed. +/- indicates if this form of the gene was positively or negatively associated with resistance. | | | | |
| --- | --- | --- | --- | --- |
| Unit | +/- | Prevalence | Pairwise comp p-value range | RefSeq accession(s) |
| *gpsB*(1)-*yoqJ* | + | 20.6% | 1.4E-14 – 6.7E-13 | WP_000200644.1-WP_000179549.1 |
| *gpsB*(2) | - | 78.8% | 1.9E-10 – 1.9E-10 | WP_000146522.1 |
| *pbp1A-recU* | + | 16.1% | 7.5E-9 – 7.5E-9 | WP_001039992.1-WP_055356909.1 |
| *cbpC* | + | 3.7% | 2.3E-10 – 2.3E-10 | WP_044813589.1 |
| hypo. prot. | + | 15.6% | 9.0E-13 – 7.1E-8 | WP_000442260.1 |

We tested sample sizes (N) ranging from 20 to 200, and for each sample size we sampled 10 random subsets of N isolates from the total dataset of 3,085 isolates. On each of these datasets we ran Scoary and required the full range of pairwise comparison p-values to be below 0.05 for a gene to be included in the results. Supplementary Table 2 shows the power at each sample size. Power is here used as the proportion of runs in which the specific gene unit was reported as significant, and the rank reflects the gene unit’s average position in the results hierarchy where detected, sorted from most to least significant. For example, the *gpsB*(1)-*yoqJ* was detected in 2/10 replicates when the sample size was 30, and on average in it placed as the fifth most significant variant in those two replicates.

| Supplementary Table 2 Power and average rank of the five identified units under a range of sample sizes, calculated as the average over 10 replicates for each sample size. | | | | | | | | | | |
| --- | --- | --- | --- | --- | --- | --- | --- | --- | --- | --- |
| N | *gpsB1-yoqJ* | | *gpsB2* | | *cbpC* | | *pbp1A-recU* | | Hypo. prot | |
|  | **Power** | **Rank** | **Power** | **Rank** | **Power** | **Rank** | **Power** | **Rank** | **Power** | **Rank** |
| 20 | 0 | - | 0 | - | 0 | - | 0 | - | 0 | - |
| 30 | 0.2 | 5 | 0.2 | 5 | 0 | - | 0 | - | 0 | - |
| 40 | 0.2 | 3.5 | 0.2 | 5 | 0 | - | 0 | - | 0.3 | 10 |
| 50 | 0.1 | 3 | 0.4 | 4.8 | 0 | - | 0 | - | 0.2 | 11.5 |
| 60 | 0.5 | 7 | 0.3 | 3.6 | 0 | - | 0.1 | 12 | 0.1 | 51 |
| 70 | 0.7 | 3.4 | 0.8 | 2.9 | 0 | - | 0 | - | 0.1 | 35 |
| 80 | 0.6 | 3 | 0.6 | 2.1 | 0.2 | 41 | 0 | - | 0.5 | 13.4 |
| 90 | 0.7 | 5.1 | 0.7 | 3.6 | 0 | - | 0 | - | 0.8 | 13.4 |
| 100 | 0.7 | 2 | 0.7 | 2.1 | 0.1 | 25 | 0.2 | 7 | 0.5 | 9 |
| 110 | 0.9 | 3.6 | 0.9 | 3.1 | 0.2 | 35 | 0.2 | 8 | 0.3 | 18.7 |
| 120 | 1 | 2.4 | 0.9 | 2 | 0.4 | 38 | 0.3 | 6 | 0.2 | 14 |
| 130 | 1 | 2.8 | 0.8 | 3.25 | 0.1 | 32 | 0.5 | 6.6 | 0.5 | 20.2 |
| 140 | 0.9 | 1.9 | 0.8 | 1.9 | 0.3 | 80.3 | 0.5 | 7.2 | 0.1 | 22 |
| 150 | 1 | 1.8 | 0.9 | 1.9 | 0 | - | 0.5 | 5.4 | 0.7 | 18.3 |
| 160 | 0.9 | 2.2 | 0.9 | 1.5 | 0.3 | 66 | 0.6 | 7.3 | 0.4 | 7.3 |
| 170 | 1 | 2.5 | 1 | 2 | 0.5 | 51.6 | 0.6 | 8.7 | 0.8 | 24.9 |
| 180 | 1 | 2.1 | 0.9 | 2.1 | 0.3 | 59.3 | 0.6 | 6 | 0.5 | 21 |
| 190 | 1 | 2.5 | 1 | 1.6 | 0.4 | 62 | 0.8 | 7.3 | 0.7 | 25.7 |
| 200 | 1 | 1.8 | 1 | 2.7 | 0.6 | 67 | 0.9 | 5.7 | 0.6 | 11.7 |

No significant genes were found at a sample size of 20. However, our results indicate that it is possible to identify causal variants with sample sizes as low as 30, where *gpsB*(1)-*yoqJ* and *gpsB*(2) was found in 20% of the runs. At a sample size of 200, Scoary always found *gpsB*(1)-*yoqJ* and *gpsB2* and these were always in the top five reported genes with an average rank of 1.8 and 2.7, respectively. *pbp1A*-*recU* was found in 90% of the replicates and had an average rank 5.7. The hypothetical protein was found in 60% of the runs at this sample size and had an average rank of 11.6. The relatively rare *cbpC* gene (with a population prevalence of only 3.7%) was detected in 60% of the runs, although with an average rank of 67.

These results indicate that while Scoary is efficient at low sample sizes, achieving high power to identify rare (such as our *cbpC* unit) or low-penetrance variants require larger samples.

1. Chewapreecha C, Harris SR, Croucher NJ, Turner C, Marttinen P, Cheng L, Pessia A, Aanensen DM, Mather AE, Page AJ *et al*: **Dense genomic sampling identifies highways of pneumococcal recombination**. *Nat Genet* 2014, **46**(3):305-309.

2. van Tonder AJ, Mistry S, Bray JE, Hill DM, Cody AJ, Farmer CL, Klugman KP, von Gottberg A, Bentley SD, Parkhill J: **Defining the estimated core genome of bacterial populations using a Bayesian decision model**. *PLoS Comput Biol* 2014, **10**(8):e1003788.

3. Land AD, Tsui HCT, Kocaoglu O, Vella SA, Shaw SL, Keen SK, Sham LT, Carlson EE, Winkler ME: **Requirement of essential Pbp2x and GpsB for septal ring closure in Streptococcus pneumoniae D39**. *Molecular microbiology* 2013, **90**(5):939-955.

4. Donati C, Hiller NL, Tettelin H, Muzzi A, Croucher NJ, Angiuoli SV, Oggioni M, Hotopp JCD, Hu FZ, Riley DR: **Structure and dynamics of the pan-genome of Streptococcus pneumoniae and closely related species**. *Genome biology* 2010, **11**(10):1.
